# Prudent planning in management of mucocele of appendix

**DOI:** 10.1016/j.ijscr.2021.105766

**Published:** 2021-03-19

**Authors:** Jallari Vyas, Mohit Badgurjar, Pankaj Saxena, Suman Parihar, Poojan Thakor

**Affiliations:** Geetanjali University, Geetanjali Medical College and Hospital, Udaipur, Rajasthan, India

**Keywords:** Mucocele, Appendix, Mucinous cystadenoma, Pseudomyxoma, Hemicolectomy

## Abstract

**Introduction:**

Appendiceal mucocele is a rare obstructive dilatation of the appendix caused by intraluminal accumulation of mucoid material. Having no typical clinical picture, patients presenting with lower right quadrant abdominal pain are sometimes mistaken for acute appendicitis. An untreated mucocele may progress leading to high mortality.

**Case report:**

A 47-year-old female presented with dull pain in the right lower abdomen, associated with generalized weakness and nausea since 6 months. Other than mild tenderness over the right iliac fossa. Physical and laboratory examination was insignificant. Abdominal sonography was doubtful between appendicular abscess and mucocele appendix. Dual contrast CT scan of abdomen was highly suggestive of an appendicular mucocele.

**Discussion:**

The patient had a vertical midline incision exploratory laparotomy done and a cystic mass measuring 7 × 4 × 4 cm^3^ was found arising from the body of appendix. With suspicion of malignancy because of size, right hemicolectomy with ileotransverse anastomosis was done. Histopathological examination showed features of low grade appendiceal mucinous neoplasm.

**Conclusion:**

Pre-operative diagnosis of appendicular mucocele is difficult, nevertheless, it is important for the selection of the appropriate surgical procedure in order to prevent intra-operative complications and early surgical intervention has excellent long-term prognosis.

## Highlights

• Mucocele of the appendix is a rare lesion of the appendix with a incidence of 0.3 to 0.7% of the appendectomies.
• Clinical presentation is usually vague and asymptomatic in quarter of patients.
• It requires keen mindset for proper evaluation.
• Prudent planning is must to avoid inadequate treatment which adds in the morbidity of patient significantly.
• And there is always risk of development of pseudomyxoma peritonei.

## 1 Introduction

Appendiceal mucocele is an obstructive dilatation of the appendix caused by intraluminal accumulation of mucoid material. It is a rare disease. The incidence is 0.2% to 0.7% of all appendectomied specimens [1]. There are 4 histologic types of appendiceal mucocele: retention cyst, mucosal hyperplasia, mucinous cystadenoma, and mucinous cystadenocarcinoma [2].

This disease does not have a typical clinical picture. Sometimes the patient has pain in the lower right quadrant of the abdomen, therefore a surgeon may confuse it for acute appendicitis. This is one of the most common surgical diseases [3]. It is important to differentiate between these two pathologies before surgery and select adequate surgical tactics. If treated improperly, the mucocele may progress, epithelial cells may escape into the peritoneal cavity, and pseudomyxoma peritonei may develop, which has a high mortality [4]. The work has been reported in line with the SCARE criteria [11].

## 2 Case report

A 47-year-old female presented with a history of right lower abdominal pain, associated with generalized weakness, nausea from last 6 months. The pain was dull, increased progressively and was relieved by simple analgesics. Her appetite was good with no appreciable changes in her weight. There was no rectal bleeding, melena, mucoid stool, dyspnea or palpitations. Her past medical histories were insignificant. She had undergone hysterectomy 10 years before. Abdominal examination revealed minor tenderness over the right iliac fossa (RIF) without localized guarding or rigidity. Digital rectal examination was normal. All routine investigations including (CEA, CA 19.9) levels were within normal ranges.

**Fig. 1:**
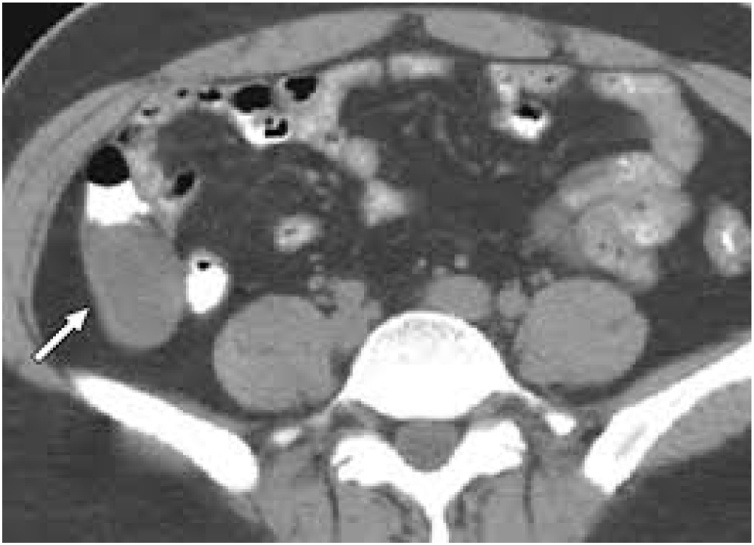
Contrast enhanced CT showing tubular mass in appendiceal area.

Abdominal sonography showed encapsulated cystic lesion in the lower quadrant of the abdomen with a liquid content of variable echogenicity? Appendicular abscess/mucocele appendix. Abdominal CT scan with oral and intravenous contrast revealed a fluid density walled off cystic lesion, measuring approx. 7.0cm × 3.8cm within the right iliac fossa in continuation with the caecum in post ileal position (Fig. 1). The wall of the lesion shows tiny specks of calcification, highly suggestive of an appendicular mucocele. The liver was normal in size and texture with no evidence of metastatic deposits.

## 3 Intervention

**Fig. 2:**
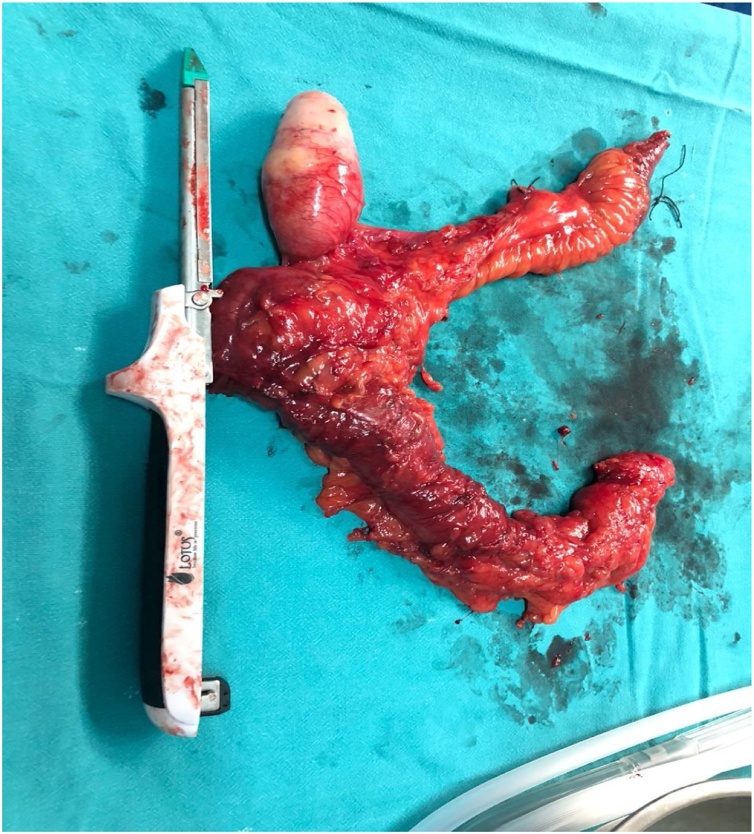
Intraoperative photograph of the resected mucocele of appendix.

With definitive impression of large mucocele of appendix, the patient underwent vertical midline incision exploratory laparotomy, which showed no evidence of peritoneal tumors, seedling or metastases. Intra operatively a cystic mass measuring 7 × 4 × 4 cm^3^ was found in the RIF arising from the body of appendix (Fig. 2). There was no evidence of perforation or abscess formation. With high suspicion of malignancy because of size of mucocele, wide base, positive lymph nodes and high chances of spillage of contents, right hemicolectomy with ileotransverse anastomosis was done. Drain was put in right paracolic gutter. Drain was removed on 3rd day. The postoperative course was uneventful and the patient was discharged on Day 6th postoperatively in a good condition. Histopathological examination showed features are of low grade appendiceal mucinous neoplasm.

## 4 Follow up

On discharge, patient was advised follow up after 15 days, 1 month and 3 months. Patient had no local or systemic complications. The patient consented in writing to the publication of the data.

## 5 Discussion

Clinical presentation of appendicular mucoceles is usually vague; furthermore, it can be asymptomatic in a quarter of patients. Most commonly, patients present with right lower quadrant pain as seen in our patient. Palpable masses have been reported in 50% of cases, whereas urinary symptoms are rare [5,6]. Pre-operative diagnosis of appendicular mucocele is difficult because of the rarity of the condition and the non-specific nature of the presenting symptoms. Nevertheless, pre-operative diagnosis is important for the selection of the appropriate surgical procedure in order to prevent intra-operative complications especially particular peritoneal dissemination [1]. Sonographic examination is considered the first-line diagnostic modality that can probably differentiate benign and malignant mucoceles [7]. An appendicular diameter of 15 mm or more has been determined as a threshold for diagnosis of mucocele with a sensitivity of 83% and a specificity of 92%. Computed tomography (CT) scan is important to confirm the diagnosis and to evaluate the extent of the disease. Fine needle aspiration cytology is not usually recommended, it increases risk of perforation and dissemination into peritoneal cavity [8].

Conventional surgery is generally preferred to laparoscopic approach as the latter increases the risk of rupture, but it is still performed for selected patients. An algorithm for the selection of the type of surgery has been formulated by Dhage-Ivatury and Sugarbaker [6,9]. Simple appendectomy is the choice for patients with benign mucocele as suggested by the presence of a normal caecum and appendicular base and no evidence of perforation. Right hemi-colectomy is recommended when malignant mucocele is suspected by the presence of a positive lymph nodes, perforated mucocele, enlarged mesenteric lymph node or a positive cytology. An accurate exploration of the abdomen is advised due to the well-known association between the appendicular mucocele and other mucin-secreting cells cancers, such as colon and ovarian cancers [10]. In conclusion, mucocele of the appendix is a rare disease with vague symptoms. Abdominal US and CT scans are important diagnostic tools, but histopathology is needed for definitive diagnosis. Surgery for benign appendicular mucoceles has an excellent long-term prognosis. This case is reported in line with the SCARE guideline.

## 6 Conclusion

Appendiceal mucocele is a rare obstructive dilatation of the appendix caused by intraluminal accumulation of mucoid material. The diagnosis of this disease proves challenging and requires a multimodal approach. The patient presented to us with a vague abdominal pain and diagnosis was established with abdominal US and CT scan, but histopathology needed for definitive diagnosis. The patient underwent right hemicolectomy with ileotransverse anastomosis was done.

## 7 Patient perspective

I am thankful to the team of surgeons for accurately detecting my problem, operating on it and treating me so I could rejoin my work with full vigor.

## Declaration of Competing Interest

Authors declare no conflict of interest.

## Sources of funding

This research did not receive any specific grant from funding agencies in the public, commercial, or not-for-profit sectors.

## Ethical approval

No ethical clearance was needed for this case report.

## Consent

Written informed consent was obtained from the patient for publication of this case report and the accompanying images.

## Author contribution

Jallari Vyas: Writing the paper.

Mohit Badgurjar: Operated, revised and edited manuscript.

Pankaj Saxena: Operated.

Suman Parihar: Operated.

## Registration of research studies

Not applicable.

## Guarantor

Dr Mohit Badgurjar.

## Provenance and peer reviewed

Not commissioned, externally peer reviewed.
